# Perspectives of School Principals on Mental Health Promotion and Prevention Among School-Aged Children—A 2025 Cross-Sectional Survey in Lodz Administrative Region in Poland

**DOI:** 10.3390/healthcare13131498

**Published:** 2025-06-23

**Authors:** Aleksandra Lewandowska, Mateusz Jankowski, Mariusz Gujski, Agata Andrzejczyk, Justyna Teliga-Czajkowska, Andrzej Silczuk

**Affiliations:** 1 Children Psychiatry Unit Specialized Psychiatric Health Care Centre in Lodz, 91-229 Lodz, Poland; 2 Department of Population Health, Centre of Postgraduate Medical Education, 01-826 Warsaw, Poland; 3 Department of Public Health, Medical University of Warsaw, 02-106 Warsaw, Poland; 4 Faculty of Health Sciences, Medical University of Warsaw, 02-109 Warsaw, Poland; 5 Department of Obstetrics and Gynecology Didactics, Faculty of Health Sciences, Medical University of Warsaw, 00-575 Warsaw, Poland; 6 Department of Environmental Psychiatry, Faculty of Life Sciences, Medical University of Warsaw, 02-353 Warsaw, Poland

**Keywords:** mental health, mental disorders, prevention, school, public policy, adolescent mental health, teachers

## Abstract

**Background:** Mental health issues among youth are a growing public health concern. Schools play a vital role in the early detection and prevention of mental health issues, with principals being central to the implementation of mental health initiatives. This study assessed the attitudes, preparedness, and practices of school principals regarding mental health promotion and prevention among school-aged children. **Methods:** A cross-sectional survey using computer-assisted web interviewing (CAWI) was conducted between March and April 2025. A 19-item questionnaire was sent to all 1564 primary and secondary school principals in the Lodz region. Data from 605 respondents (response rate: 38.7%) were analyzed. **Results:** A total of 94.9% of the principals reported an increase in student mental health problems over the past five years. Over one-quarter of school principals (26.3%) declared a very good or rather good level of preparation for teachers to work with students diagnosed with a mental disorder. Moreover, 42.3% declared a very good or rather good level of preparation of teachers for conversations with parents about mental health problems observed in their children. Mental health education was conducted in 94.7% of schools, and teacher training in 73.2% of schools. Secondary schools more often offered such training (81.9% vs. 70.7%; *p* = 0.01) and reported stronger support from specialist teachers (79.7% vs. 67.7%; *p* = 0.01) than primary schools did. Rural schools rated teacher-parent communication more positively (47.0% vs. 37.7%; *p* = 0.02) despite fewer training initiatives (68.7% vs. 77.7%; *p* = 0.01). Suicide response procedures were implemented in 78.5% of schools. **Conclusions:** The findings confirm a marked rise in student mental health needs and reveal structural disparities in providing school-based support. Enhancing school leadership, expanding teacher training, and ensuring systemic support may facilitate mental health promotion interventions in schools.

## 1. Introduction

Mental health issues among children and adolescents are becoming a growing public health problem worldwide [1,2]. The World Health Organization indicates that in 2023, one in five children and adolescents may suffer from mental or behavioral disorders, and suicide is the fourth leading cause of death among adolescents worldwide [1]. Kieling et al. (2024) confirmed the high burden of mental health problems in children and adolescents using data from the Global Burden of Disease Study [2]. The prevalence of mental health problems increased from 6.80% in children aged 5–9 years to 12.40% among those aged 10–14 years and up to 13.96% in adolescents aged 15–19 years [2]. The incidence of individual disorders varies depending on age group, and gender-specific patterns change with age [2]. It is estimated that over a third of mental health issues occur in children before the age of 14, yet many of these cases remain undiagnosed and untreated, with significant negative impacts on the young person and their family, as well as on communities, civil society, and the economy [3]. Early intervention and preventive strategies are crucial because mental health issues that emerge in early life often persist into adulthood, affecting education, employment, and quality of life [4].

Schools, as the environment of the everyday functioning of children and adolescents, play a fundamental role in promoting mental health and providing early support. Students spend about one-third of their day at school, which makes this environment an important place for the development of cognitive, emotional, and social competencies [5]. Schools provide unique opportunities for the early identification of mental health issues, psychoeducation, and the implementation of universal prevention programs [6]. Moreover, the school environment has a significant impact on students’ emotional well-being, mental resilience, and ability to establish positive social interactions. Given their daily contact with a large group of students, teachers are in a unique position to monitor and detect the symptoms of mental disorders [7]. Teachers can effectively detect early signs of mental health disorders if they have adequate knowledge about the symptoms and their recognition. An attitude towards mental health issues based on openness, mindfulness, and readiness to provide support facilitates faster referral of students to specialist help, which in turn increases the chances of early diagnosis and effective treatment [8].

Teachers and school staff often act as the first observers, able to detect the early signs of emotional and behavioral difficulties in students [9]. However, their effectiveness depends on appropriate training, institutional support, and clearly defined procedures [10]. Available studies indicate that teachers who refer students to mental health services have a higher level of awareness of mental disorders in children [11]. Low levels of knowledge about psychiatric problems significantly reduce teachers’ willingness to provide support or refer students to specialist help [11].

Schools are identified as strategic locations for implementing preventive interventions in many high-income countries and some low- and middle-income countries [12]. Mental health interventions implemented in school settings include developing competencies in achieving and maintaining positive mental health, reducing the stigma of mental disorders, increasing knowledge about mental illness and available treatments, and supporting intentions and behaviors related to seeking help, coping with stress, and strengthening mental resilience [13].

Despite some doubts about the effectiveness of schools as places for implementing health interventions [14], available evidence indicates that teachers worldwide are generally open to developing their mental health competencies, which allows them to effectively and responsibly respond to the needs of young people who present with concerning symptoms [15].

School principals, as institutional leaders and managers, play a key role in setting priorities, coordinating mental health activities, and building supportive school climates [16]. Their attitudes towards mental health promotion, knowledge of preventive practices, and perceived barriers and facilitators significantly shape the implementation of health strategies in schools [17]. Their knowledge, attitudes, and skills in identifying barriers and facilitators to the implementation of preventive actions are critical for the success of school initiatives. Approaches to mental health literacy in education are considered fundamental to mental health promotion, prevention, and care and are well-validated in the context of adolescent mental health [18]. Despite the key role of principals, their perspectives remain under-researched, especially in Central and Eastern Europe. To date, research has focused mainly on teachers, with limited analysis of the leadership dynamics influencing school health initiatives [19]. Obtaining data directly from principals provides a valuable opportunity to better understand the systemic challenges and potential facilitators of improving mental health support in education.

This study aimed to characterize the attitudes of school principals toward mental health promotion and prevention among school-aged children.

## 2. Materials and Methods

### 2.1. Study Design and Population

In this cross-sectional survey, data were collected over four weeks, from 14 March to 11 April 2025, using the computer-assisted web interviewing (CAWI) method. The survey was administered online through a dedicated questionnaire available via Google Forms.

The study targeted all 1564 school principals from the Lodz region, located in central Poland, and included principals from primary schools (serving students aged 7–15) and secondary schools, which in the Polish educational system include general high schools, technical secondary schools, and vocational secondary schools (serving students aged 15–19). The Lodz Provincial Board of Education supported the dissemination of the survey link to school principals, which facilitated communication about the study through its official information system.

Following recent educational reforms, primary education in Poland now extends until the age of 15, and secondary education has diversified into general academic, technical, and vocational tracks. Each participant received an individualized link to the survey, which was distributed via the regional educational information system. Two email reminders containing information about the study’s purpose and an invitation to participate were sent to all principals during the data-collection period. The invitation was sent to all 1564 school principals (both public and private) in the Lodz region. Lodz is one of Poland’s major administrative regions, covering approximately 18,219 km^2^ with a population of about 2.4 million [20]. The region exhibits a balanced mix of urban and rural areas and reflects demographic and socioeconomic characteristics representative of the broader national population, supporting the generalizability of our findings. A proprietary questionnaire was developed for this study based on an extensive review of the current international and national literature related to the research topic.

### 2.2. Study Questionnaire

The study questionnaire was prepared based on a literature review and adjusted to the current organization of the children’s and adolescents’ mental health and education systems in Poland (Supplementary File S1). Mental health specialists and public health specialists worked on the questionnaire. Moreover, the Lodz Provincial Board of Education—a public institution tasked with the supervision of educational facilities–was asked for a review, in particular, the way the questions were formulated in the context of the school’s daily activities and the assessment of the risk of failure to respond due to the questions’ interference with the privacy and anonymity of respondents.

The study questionnaire included 19 questions regarding mental health promotion and prevention among school-aged children. Questions regarding personal characteristics were also addressed. A pilot study was carried out in which 6 school principals filled out the questionnaire twice, 5 days apart. After the pilot, 1 question was removed and 2 questions were changed. The study protocol was reviewed and approved by the Ethics Commission of the Medical University of Warsaw (approval number: AKBE/40/2025 as of 24 February 2025).

### 2.3. Data Analysis

The responses received from the school principals were coded into an electronic database. IBM SPSS Statistics version 29 was used for data analysis. Variables were presented as frequencies and proportions. The Chi-squared test was used to compare differences between categorical variables. The statistical significance level was set at *p* < 0.05.

## 3. Results

Data were received from 605 respondents, with a response rate of 38.7%. Among the respondents, 81.8% were females, one-third (34.5%) had less than 5 years of professional experience as a school principal, 77.2% managed the primary school, and 49.6% worked in schools located in rural areas (Table 1).

Most of the school principals (*n* = 94.9%) agreed that the number of mental health problems among children and adolescents that affect their functioning at school has increased over the last five years (Table 2). Over one-quarter of school principals (26.3%) declared a very good or rather good level of preparation of teachers to work with students diagnosed with a mental disorder at the school they manage, wherein 15.2% rated this preparation level as rather bad or very bad. Among the school principals, 42.3% declared a very good or rather good level of preparation of teachers for conversations with parents about mental health problems observed in their children, and only 9.9% considered this preparation level as rather bad or very bad. Implementation of recommendations issued by specialists in the psychiatric care system for children and adolescents in schools was rated as good or very good by 53.1% of school principals. Among the school principals (70.4%) declared a rather good or very good level of support that a specialist teacher in the school they manage provides to a student diagnosed with a mental disorder (Table 2). Lessons on mental health for students were organized in 94.7% of schools, 73.2% of schools organized training for teachers on working with children diagnosed with mental disorders, and 78.5% of schools had procedures in place to deal with students who attempted suicide (Table 2).

Training for teachers on working with children diagnosed with mental disorders (Table 3) was more often organized in secondary (81.9%) than in primary schools (70.7%; *p* = 0.01), as well as in schools located in urban (77.7%) than in rural areas (68.7%; *p* = 0.01). There were no sociodemographic differences when related to organization lessons on mental health for students and procedures in place to deal with a student who attempts suicide (Table 3).

School principals (Table 4) who managed schools in urban areas compared to principals from rural schools more often observed a rise in mental health problems over the last five years (97% vs. 92.7%; *p* = 0.02). However, school principals from rural areas more often declared rather good or very good preparation of teachers for conversations with parents about mental health problems in children (47.0% vs. 37.7%; *p* = 0.02). Those who managed secondary schools, compared to those from primary schools, more often declared a rather good or very good level of support that a specialist teacher provides to a student with a mental disorder (79.7% vs. 67.7%; *p* = 0.01).

## 4. Discussion

This is the first cross-sectional survey to assess the attitudes of school principals toward mental health promotion and prevention among school-aged children. The findings from of this study showed that school principals noticed an increasing incidence of mental health issues among school-aged children. This study was carried out in 2024, after the organizational changes in the educational system in 2017 and the implementation of a new model of psychiatric care in previous years. School principals declared a moderate level of preparedness to work with students diagnosed with mental disorders. However, most schools implemented policies aimed at preparing teachers to support students with mental health issues. Gaps in the training of teachers on mental health issues and procedures in place to deal with students who attempt to commit suicide were identified. School type (primary or secondary) and location of school impacted selected attitudes toward mental health promotion and prevention among school-aged children.

In this study, 94.9% of respondents stated that the number of mental problems among students had increased in the last five years. This phenomenon was observed regardless of gender, type of institution, or seniority of the principal, which indicates its universal nature. The observed increase in problems concerns the entire school population, both in cities and rural areas, in primary and secondary schools. These data are consistent with global trends indicating a deterioration in the mental well-being of young people, especially since the COVID-19 pandemic, due to social isolation, distance learning, and increased stress [21,22]. Considering these results, the mental health of children and adolescents appears to be one of the key challenges for education and healthcare systems, requiring coordinated institutional action. The level of preparation of teachers for working with students with psychiatric diagnoses is moderate—only 26.3% of principals assessed it as good or very good, and 58.5% as average. Many teachers also do not feel confident in talking to parents about children’s mental problems—47.8% indicated a moderate level and only 42.3% considered their preparation to be good or very good. These results are consistent with previous studies, which indicate a lack of appropriate competence, knowledge, and tools to support students in mental crises [7,11,15]. The findings from this study showed that schools in Poland undertake numerous activities to promote mental health—94.7% organize educational activities for students, 73.2% conduct training for teachers, and 78.5% have implemented procedures for responding to suicide attempts. This study indicates a growing awareness of this problem. At the same time, there are significant inequalities in access to these activities: secondary schools are more likely to organize training (81.9%) than primary schools (70.7%), and urban schools are less likely to report difficulties than rural schools (77.7% vs. 68.7%). This is probably due to the different availabilities of resources, specialists, and institutional support [6,23].

The findings of this study showed that most principals noticed an increase in students’ mental health problems —97.0% in urban schools and 92.7% in rural schools (*p* = 0.02). At the same time, rural school principals more often assess teachers as better prepared for conversations with parents (47.0% vs. 37.7%; *p* = 0.02), which may result from closer relationships in smaller communities. Rural-urban differences in the organization of mental health support remain a significant public health. The findings of this study are generally consistent with previously published data. Rural schools, although characterized by more direct relationships in the local community, often do not have access to sufficient institutional support, which makes it difficult to respond systemically to students’ mental health problems [18,24]. Moreover, in secondary schools, the level of support offered by specialist teachers was higher (79.7% vs. 67.7%; *p* = 0.01), and staff training was more frequent (81.9% vs. 70.7%; *p* = 0.01). No differences were found in the organization of lessons for students or the existence of intervention procedures in the event of a suicide attempt. These data show that older students have relatively better access to support, which may be due to the more formalized approach in secondary schools, greater experience of staff working with young people, and a higher level of cooperation with external institutions. In contrast, primary school students are often in a more sensitive developmental period and require intensive support. These differences indicate the need to cover primary schools with special care in the implementation of prevention and intervention programs and to equalize support standards at all stages of education.

The results of studies available in the scientific literature indicate the role of the school principal as a key leader in the promotion of mental health [16]. Schools whose principals actively support activities are more likely to implement training and intervention procedures [25]. This confirms previous reports from the literature and indicates the need to develop leadership competencies in management staff [16,25,26]. Models such as the Interconnected Systems Framework (ISF) emphasize the need for long-term planning, interdisciplinary cooperation, and data-based leadership [25,26]. However, in Polish conditions, there is a significant gap in the preparation of principals to fulfill this role, both in terms of crisis management and strategic planning of preventive actions [27]. They often rely on intuition and personal experience without access to specialist tools or formal system support. Effective intervention requires not only the will to act but also professional competencies in team building, coordination with external institutions, and resource management. Therefore, it is necessary to implement development programs for school management staff—both in the form of training, mentoring, and expert support—that will allow principals to effectively perform the role of mental health leaders. Without their active and competent involvement, even the best-designed preventive actions have limited chances of lasting and effective implementation.

This study has the limitations typical of cross-sectional surveys. The questionnaire was self-prepared, and the scope of the analysis was limited to data collected in the short survey. Recall bias may have occurred. This study was limited to school principals from one administrative region in Poland (Lodz voivodeship); however, all school principals were invited, and the response rate was relatively high. Moreover, the scope of the statistics was limited to basic statistics, as the number of collected variables did not allow for advanced regression analyses. This study provides self-reported survey responses; therefore, the results might not truly reflect the teachers’ or schools’ preparedness but rather the reflection of principals’ attitudes.

This study has practical implications for public policy. The data presented in this study may be used to shape mental health policies in Poland. Moreover, gaps in the implementation of mental health prevention initiatives in schools can be used to plan and implement public measures to strengthen mental health services in schools. This study also highlights the role of schools in mental health prevention and promotion.

## 5. Conclusions

The results of the study confirm the growing scale of mental problems among students and reveal serious gaps in the preparation of teachers to work with children diagnosed with mental disorders and cooperate with their parents. Although schools undertake valuable preventive initiatives, their effectiveness is limited by structural inequalities, especially between urban and rural institutions. The role of the principal as a leader in mental health activities is crucial—their active involvement significantly increases the chances of success of the programs. Systemic strategies that consider the local context and support the development of staff competencies are needed to enable schools to support the mental well-being of children and young people. The results are in line with European priorities in the field of mental health and can provide a basis for shaping coherent policies in this area.

## Figures and Tables

**Table 1:** Characteristics of the study population (*n* = 605).

Gender	*n*	%
female	495	81.8
male	110	18.2
Years of experience as a school principal		
1–5	209	34.5
6–10	115	19.0
11–20	113	18.7
21–30	100	16.5
over 30 years	68	11.2
Type of school		
primary school	467	77.2
general secondary school	67	11.1
technical school	60	9.9
vocational school	11	1.8
Location of the school		
rural area	300	49.6
small city (<20,000 residents)	70	11.6
medium city (20,000–100,000 residents)	138	22.8
big city (over 100,000 residents)	97	16.0

**Table 2:** School principals’ opinions on mental health promotion and prevention among school-aged children (*n* = 605).

Variable	*n*	%
In your opinion, has the number of mental health problems among children and adolescents that affect their functioning at school increased over the last 5 years?		
definitely yes	361	59.7
rather yes	213	35.2
rather no	26	4.3
definitely no	3	0.5
I do not know/difficult to tell	2	0.3
How would you rate teachers’ preparedness to work with students diagnosed with mental disorders?		
definitely good	11	1.8
rather good	148	24.5
moderate	354	58.5
rather bad	84	13.9
definitely bad	8	1.3
How would you rate teachers’ preparedness to discuss students’ mental health concerns with parents?		
definitely good	20	3.3
rather good	236	39.0
moderate	289	47.8
rather bad	58	9.6
definitely bad	2	0.3
How do you assess the degree of implementation of recommendations issued by specialists in the psychiatric care system for children and adolescents in the school you manage?		
definitely good	29	4.8
rather good	292	48.3
moderate	248	41.0
rather bad	30	5.0
definitely bad	6	1.0
How would you rate the level of support that a specialist teacher in the school you manage provides to a student diagnosed with a mental disorder?		
definitely high	81	13.4
rather high	345	57.0
moderate	162	26.8
rather low	16	2.6
definitely low	1	0.2
Does your school organize lessons on mental health for students?		
yes	573	94.7
no	32	5.3
Does your school organize training for teachers on working with children diagnosed with mental disorders?		
yes	443	73.2
no	162	26.8
Does your school have procedures in place to deal with a student who attempts suicide?		
yes	475	78.5
no	130	21.5

**Table 3:** Interventions undertaken in schools to promote mental health and prevent mental health problems among school-aged children (*n* = 605). Statistically significant values are bolded (*p* < 0.05).

Variable	Does Your School Organize Lessons on Mental Health for Students? (Yes)		Does Your School Organize Training for Teachers on Working with Children Diagnosed with Mental Disorders? (Yes)		Does Your School Have Procedures in Place to Deal with a Student Who Attempts Suicide? (Yes)	
	*n* (%)	*p*	*n* (%)	*p*	*n* (%)	*p*
Gender						
female (*n* = 495)	470 (94.9)	0.6	363 (73.3)	0.9	383 (77.4)	0.1
male (*n* = 110)	103 (93.6)		80 (72.7)		92 (83.6)	
Years of experience as a school principal						
1–5 (*n* = 209)	195 (93.3)	0.7	146 (69.9)	0.8	163 (78.0)	0.8
6–10 (*n* = 115)	111 (96.5)		87 (75.7)		87 (75.7)	
11–20 (*n* = 113)	108 (95.6)		84 (74.3)		88 (77.9)	
21–30 (*n* = 100)	94 (94.0)		75 (75.0)		81 (81.0)	
over 30 years (*n* = 68)	65 (95.6)		51 (75.0)		56 (82.4)	
Type of school						
primary school (*n* = 467)	440 (94.2)	0.3	330 (70.7)	**0.01**	359 (76.9)	0.07
secondary school (*n* = 138)	133 (96.4)		113 (81.9)		116 (84.1)	
Location of the school						
rural area (*n* = 300)	288 (96.0)	0.2	206 (68.7)	**0.01**	227 (75.7)	0.09
urban area (*n* = 305)	285 (93.4)		237 (77.7)		248 (81.3)	

**Table 4:** Sociodemographic differences in school principals’ opinions on mental health promotion and prevention among school-aged children (*n* = 605). Statistically significant values are bolded (*p* < 0.05).

Variable	Rise in Mental Health Problems (Rather Yes/Definitely Yes) over the Last 5 Years		Teachers’ Preparedness to Work with Students Diagnosed with Mental Disorders (Rather Good or Very Good)		Teachers’ Preparedness to Discuss Students’ Mental Health Concerns with Parents (Rather Good or Very Good)		Degree of Implementation of Recommendations Issued by Specialists in the Psychiatric Care System for Children (Rather Good or Very Good)		Level of Support That Specialist Teacher Provides to a Student with Mental Disorder (Rather Good or Very Good)	
	*n* (%)	*p*	*n* (%)	*p*	*n* (%)	*p*	*n* (%)	*p*	*n* (%)	*p*
Gender										
female (*n* = 495)	470 (94.9)	0.9	124 (25.1)	0.1	205 (41.4)	0.3	254 (51.3)	0.07	344 (69.5)	0.3
male (*n* = 110)	104 (94.5)		35 (31.8)		51 (46.4)		67 (60.9)		82 (74.5)	
Years of experience as a school principal										
1–5 (*n* = 209)	193 (92.3)	0.1	66 (31.6)	0.1	95 (45.5)	0.4	126 (60.3)	0.1	156 (74.6)	0.09
6–10 (*n* = 115)	114 (99.1)		28 (24.3)		42 (36.5)		52 (45.2)		78 (67.8)	
11–20 (*n* = 113)	107 (94.7)		28 (24.8)		53 (46.9)		58 (51.3)		82 (72.6)	
21–30 (*n* = 100)	95 (95.0)		26 (26.0)		41 (41.0)		51 (51.0)		71 (71.0)	
over 30 years (*n* = 68)	65 (95.6)		11 (16.2)		25 (36.8)		34 (50.0)		39 (57.4)	
Type of school										
primary school (*n* = 467)	441 (94.4)	0.4	120 (25.7)	0.5	202 (43.3)	0.4	242 (51.8)	0.3	316 (67.7)	**0.01**
secondary school (*n* = 138)	133 (96.4)		39 (28.3)		54 (39.1)		79 (57.2)		110 (79.7)	
Location of the school										
rural area (*n* = 300)	278 (92.7)	**0.02**	87 (29.0)	0.1	141 (47.0)	**0.02**	158 (52.7)	0.8	212 (70.7)	0.9
urban area (*n* = 305)	296 (97.0)		72 (23.6)		115 (37.7)		163 (53.4)		214 (70.2)	

## Data Availability

The raw data supporting the conclusions of this article will be made available by the authors upon request.

## Supplementary Materials

The following supporting information can be downloaded at https://www.mdpi.com/article/10.3390/healthcare13131498/s1, Supplementary File S1. Study Questionnaire.
