# Supplementary material for: Perspectives of School Principals on Mental Health Promotion and Prevention Among School-Aged Children—A 2025 Cross-Sectional Survey in Lodz Administrative Region in Poland
Source: Healthcare (Basel). 2025 Jun 23;13(13):1498. doi: 10.3390/healthcare13131498 (PMC12250504; doi:10.3390/healthcare13131498)
Supplement: Supplementary file 1 [file healthcare-13-01498-s001.zip › healthcare-3699998-supplementary.pdf]

Survey Questionnaire

Study Title: *Attitudes of School Principals in the Łódź Voivodeship Toward the Prevention of Mental Health Disorders in Children and Adolescents*

This research project is conducted as part of internal research at the Department of Community Psychiatry, Faculty of Health Sciences, Medical University of Warsaw, in collaboration with Dr. Aleksandra Lewandowska, National Consultant in Child and Adolescent Psychiatry.

The study aims to explore and better understand knowledge and attitudes regarding mental health disorders in children and adolescents, as well as the role of schools in mental health prevention. The findings will provide an evidence base for shaping public health policy. Results will be used to prepare scientific publications and may contribute to the development of mental health initiatives in Poland.

Please note that all data will be collected anonymously. By completing the questionnaire, you consent to the use of anonymized data for the purposes of the above-mentioned research.

Questions:

1. **Gender:**

    Female

    Male

2. **Years of experience as a school principal:**

    1–5 years

    6–10 years

    11–20 years

    21–30 years

    More than 30 years

3. **Type of school:**

    Primary school

    General secondary school (liceum)

    Technical secondary school (technikum)

    First-level vocational school

    Second-level vocational school

4. **School location:**

    Large city (over 100,000 inhabitants)

Medium-sized city (20,000–100,000 inhabitants)

Small town (under 20,000 inhabitants)

Rural area

5. **In your opinion, has the number of mental health issues affecting school functioning among children and adolescents increased over the past 5 years?**

   Definitely yes

   Probably yes

   Probably not

   Definitely not

   No opinion

6. **How prepared are teachers at your school to work with students diagnosed with mental disorders?**

   Very low

   Low

   Moderate

   High

   Very high

7. **How well are recommendations from child and adolescent mental health professionals implemented at your school?**

   Very poorly

   Poorly

   Moderately

   Well

   Very well

8. **How prepared are teachers to talk with parents about students' mental health concerns?**

   Very low

   Low

   Moderate

   High

Very high

9. **How would you rate specialist support for teachers working with students with mental health diagnoses?**

Insufficient

Poor

Adequate

Good

Excellent

10. **Does your school provide teacher training on mental disorders?**

Yes

No

If yes, what are the sources of training? (select all that apply)

Organized by the school

EU-funded projects

Public education system

NGOs

Other external institutions (e.g., universities, counseling centers)

11. **Does your school have procedures for suicide attempts??**

Yes

No

Since 2020, a three-tier model of psychiatric care for children and adolescents has been implemented in Poland, based, among other elements, on cooperation with educational institutions to support the child/adolescent.

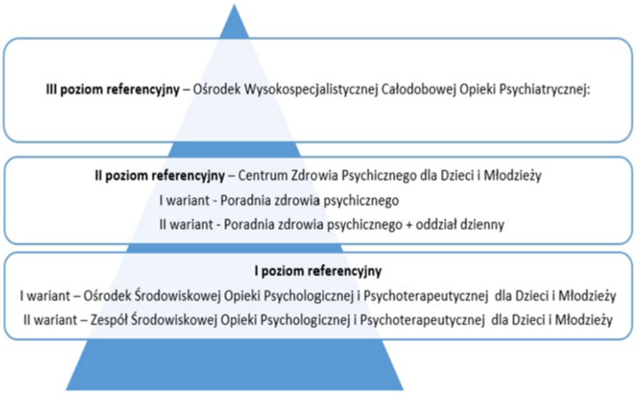

Prepared by the Department of Public Health, Ministry of Health

12. How would you assess the level of knowledge about the new model of psychiatric care for children and adolescents among staff at your school?

Very low

Low

Moderate

High

Very high

13. How would you rate your school's cooperation with Tier I services (Community Psychological and Psychotherapeutic Care Centers)?

Very poor

Poor

Adequate

Good

Very good

14. How would you rate cooperation with Tier II services (Mental Health Outpatient Clinics for Children and Adolescents)?

Very poor

Poor

Adequate

Good

Very good

15. How would you rate cooperation with Tier II services (Day Psychiatric Wards for Children and Adolescents)?

Very poor

Poor

Adequate

Good

Very good

16. How would you rate cooperation with Tier III services (Inpatient Psychiatric Wards)?

Very poor

Poor

Adequate

Good

Very good

17. Are any evidence-based mental health prevention programs recommended by the National Center for Addiction Prevention (source: https://programyrekomendowane.pl/) implemented at your school?

Yes

No

If yes, how many such programs are implemented per year?

Fewer than 5

6–10

More than 10

18. Are there any mental health education lessons provided to students at your school?

Yes

No

If yes, who conducts them? (select all that apply)

Homeroom teacher

Specialist teacher

Other teacher

Non-governmental organizations

Other external institutions (e.g., universities, counseling centers)

19. If legislation in Poland allowed for banning mobile phone use in schools, would you support such a ban at your school?

Definitely yes

Probably yes

Probably not

Definitely not

No opinion

If yes, do you believe such a ban would help students develop social skills?

Definitely yes

Probably yes

Probably not

Definitely not

No opinion
